# Motivational and psychological correlates of bodybuilding dependence

**DOI:** 10.1556/JBA.3.2014.3.6

**Published:** 2014-09-27

**Authors:** NEIM N. EMINI, MALCOLM J. BOND

**Affiliations:** School of Medicine, Flinders University, Adelaide, Australia

**Keywords:** stress, anger, hostility, aggression

## Abstract

*Background and aims*: Exercise may become physically and psychologically maladaptive if taken to extremes. One example is the dependence reported by some individuals who engage in weight training. The current study explored potential psychological, motivational, emotional and behavioural concomitants of bodybuilding dependence, with a particular focus on motives for weight training. Using a path analysis paradigm, putative causal models sought to explain associations among key study variables. *Methods*: A convenience sample of 101 men aged between 18 and 67 years was assembled from gymnasia in Adelaide, South Australia. Active weight trainers voluntarily completed a questionnaire that included measures of bodybuilding dependence (social dependency, training dependency, and mastery), anger, hostility and aggression, stress and motivations for weight training. *Results:* Three motives for weight training were identified: mood control, physique anxiety and personal challenge. Of these, personal challenge and mood control were the most directly salient to dependence. Social dependency was particularly relevant to personal challenge, whereas training dependency was associated with both personal challenge and mood control. Mastery demonstrated a direct link with physique anxiety, thus reflecting a unique component of exercise dependence. *Conclusions:* While it was not possible to determine causality with the available data, the joint roles of variables that influence, or are influenced by, bodybuilding dependence are identified. Results highlight unique motivations for bodybuilding and suggest that dependence could be a result of, and way of coping with, stress manifesting as aggression. A potential framework for future research is provided through the demonstration of plausible causal linkages among these variables.

## Introduction

Even rewarding activities have an addiction potential (Allegre, Souville, Therme & Griffiths, 2006; Terry, SzaboGriffiths, 2004). Exercise, for example, may become maladaptive if taken to extremes (Ackard, Brehm & Steffen, 2002; Hall, Kerr, Kozub & Finnie, 2007; Hamer & Karageorghis, 2007). Indicators of maladaptive exercise include working out several times a day, or for longer periods than recommended, obsessing over calories expended, anger if interrupted, cancelling or avoiding social or occupational responsibilities in order to exercise, and centring daily schedules around exercise (Adams & Kirkby, 2002; Terry et al., 2004). Terms applied to such behaviour include exercise dependence (Hamer & Karageorghis, 2007; Hausenblas & Symons Downs, 2002), over-exercising (Long, Smith, Midgley & Cassidy, 1993), heightened commitment to exercise (Yates, Edman, Grago & Crowell, 2001), obligatory exercise (Ackard et al., 2002; Hall et al., 2007), and exercise addiction (Annett, Cripps & Steinberg, 1995; Mathers & Walker, 1999). Importantly, exercise dependence is considered distinct from the behaviour of the ‘gym enthusiast’ by the symptoms of tolerance, withdrawal, lack of control, intention efforts, time, reduction in other activities, and continuance evident in the dependent individual (Berczik et al., 2012; de Coverley Veale, 1987; Freimuth, Moniz & Kim, 2011).

Exercise dependence has long been studied with reference to compulsive running among athletes (Chapman & DeCastro, 1990; Freimuth et al., 2011; Hall et al., 2007; Ogden, Veale & Summers, 1997) and in relation to eating disorders among women (Ackard et al., 2002; Mond, Hay, Rodgers & Owen, 2006). It can be a secondary addiction to an eating disorder, or occur without an eating disorder as a primary addiction. Generally, weight and shape concerns are lower in those with primary exercise dependence, as the purpose of exercise, when an eating disorder is present, is almost exclusively for weight and shape control (Freimuth et al., 2011).

Smith, Hale and Collins (1998) proposed that exercise dependence among bodybuilders is a specific exemplar of this behaviour. While weight training may be initiated for innocent reasons such as improving strength and fitness, over time it may take on an exaggerated importance. This is particularly likely in situations where physique and self-esteem are intertwined (Hamer & Karageorghis, 2007). Research concerning bodybuilding dependence has focused on socio-demographic, motivational and psychological correlates. For example, Smith et al. (1998) and Hurst, Hale, Smith and Collins (2000) determined that dependent persons were more likely to begin weight training to improve self-esteem. Stronger, leaner, muscular males, for example, may be per ceived as socially dominant, and more likely than their smaller counterparts to receive respect and admiration from their peers, which may foster an obsession with bodybuilding (Cafri, Van den Berg & Thompson, 2006; Smith & Stewart, 2012). Hurst et al. (2000) further noted that the social atmosphere and support gained through weight training were associated with dependence.

Psychologically, dependence has been linked to both muscle dysmorphia (Cafri et al., 2006; Giardino & Procidano, 2012; Smith & Hale, 2004) and lower life satisfaction (Smith & Hale, 2005). Further, a high drive for muscularity has been associated with the susceptibility to other addictions (Hale, Roth, DeLong & Briggs, 2010). Finally, bodybuilding dependence has been shown to be more likely among persons from lower socioeconomic backgrounds and those not currently involved in a romantic relationship (Smith & Hale, 2005). Given the growing literature concerning this phenomenon, the current study explored potential psychological, motivational, emotional and behavioural concomitants of bodybuilding dependence, with a focus on anger, hostility and aggression.

### Anger, hostility and aggression

Anger, hostility and aggression reflect the emotional, cognitive and behavioural dimensions of a personality construct often subsumed under the generic term hostility (Smith, Glazer, Ruiz & Gallo, 2004). The three dimensions are related in that hostility and anger may create an action tendency to engage in physical or verbal aggression (Barefoot & Lipkus, 1994). Depending on individual coping styles this action tendency may either be outwardly expressed or suppressed (Smith et al., 2004). The transactional model discussed by Smith et al. suggests that manifest aggression towards significant others may elicit antagonistic responses that may lead to stress, higher levels of hostility and anger, and ultimately the perpetuation of aggression in an ongoing feedback cycle.

Based on personal experience and interviews with fitness trainers, Gavin (2004) noted that bodybuilders tended to have aggressive personalities, a characteristic that may be further reinforced by the use of steroids (Giardino & Procidano, 2012). However, bodybuilders were often motivated by the desire to rid themselves of aggressive tendencies (Crossley, 2005). Indeed, those addicted to exercise were often more restless and stressed prior to exercise (Anshel, 1991). Thus parallels can be identified between bodybuilding dependence and hostility; both can result in a lack of social support which in turn may manifest as stress (Gallo & Smith, 1999; Hart, 1999; Smith et al., 2004), vulnerable levels of self-esteem, and increased levels of vigorous activity (Gallo & Smith, 1999; Hart, 1999; Musante, Treiber, Davis, Strong & Levy, 1992; Smith et al., 2004; Smith & Hale, 2005). Further, in his ethnographic study, Klein (1993) argued bodybuilding to be an important aspect of gender display, of which appearance is one element. Munroe-Chandler, Kim and Gammage (2004) noted appearance imagery during exercise was more frequent than technique or energy imagery. Aggressive behaviour to assert social dominance and masculine identity could also play a role in this.

Observations such as these suggest the value of examining associations between bodybuilding dependence, anger, hostility and aggression. Also, they suggest that constructs such as stress and motives for bodybuilding might contribute to the understanding of dependence. The aim of the current study was therefore to explore associations among these constructs, while also testing previous findings that dependence is associated with demographic variables and relationship status. While it was not possible to address causal links, the contributions of variables that may influence bodybuilding dependence were examined, thus allowing causal hypotheses to be framed for future research. In particular, the contributions to bodybuilding dependence of motives, over and above those made by stress, anger, hostility and aggression were examined.

## Methods

### Participants and procedure

Eligible participants were men attending one of eight gymnasia in Adelaide, South Australia. Questionnaires were distributed with permission of the centres’ managers. Eligible participants were those who self-identified as current regular weight trainers. A verbal briefing and written summary of the aims and requirements of the study was provided, with the voluntary nature of the study and confidentiality of responses emphasised. A prepaid envelope was provided for the return of the questionnaire. The estimated return rate was 65%. Data analyses were conducted using SPSS (Version 17).

### Instruments

The questionnaire comprised the following scales and also sought demographic information (age, education, relationship status) and details of weight training history.

#### Bodybuilding Dependence Scale (BDS)

Symptoms of dependence are quantified (Smith et al., 1998) using acknowledged criteria (Morgan, 1979; Veale, 1995). Participants indicate how often each item is true for them (1 = ‘never’, 7 = ‘very often’). Three subscales are computed (Hurst et al., 2000; Munroe-Chandler et al., 2004; Smith & Hale, 2004, 2005; Smith et al., 1998): social dependency (4 items; range 4-28; ‘In the event of a conflict between my weight training and my job, my training would always come first’), training dependency (3 items; range 3-21; e.g., ‘I will not miss a scheduled weight training workout, even if I do not feel like training’) and mastery (2 items; range 2-14; e.g., ‘I weight train even when I have a cold or flu’). Higher scores denote greater dependence.

#### Aggression Questionnaire

Verbal and physical aggression, anger and hostility are assessed (Buss & Perry, 1992) using items such as ‘I tell my friends openly when I disagree with them’ (verbal aggression, 5 items, range 5-25), ‘Once in a while I cannot control the urge to strike another person’ (physical aggression, 9 items, range 9-45), ‘I sometimes feel like a powder keg ready to explode’ (anger, 7 items, range 7-35), and ‘I am suspicious of overly friendly strangers’ (hostility, 8 items, range 8-40). Participants indicate how characteristic items are of them (1 = ‘extremely uncharacteristic’, 5 = ‘extremely characteristic’), with higher scores indicating higher levels of each construct.

#### Perceived Stress Scale (PSS)

The 10-item version (Cohen & Williamson, 1988) of the PSS was used to provide an appraisal of day-to-day stress. The degree to which participants report their lives to be unpredictable and overloaded (Cohen, Kamarck & Mermelstein, 1983) is quantified by responses to such items as ‘felt nervous and stressed’ and ‘felt that things were going your way’ (1 =‘never’, 5 = ‘very often’). A higher score (range 10-50) suggests greater stress.

#### Motives for weight training

Using a 5-point scale, participants noted the degree to which 21 motives applied to them. Motives were derived from relevant literature (Crossley, 2005; Gavin, 2004) and the personal observations of the first author. Items surveyed such domains as the psychological (e.g., ‘to increase self-confidence’), physical (e.g., ‘to put on muscle mass’), social (e.g., ‘for social contact’), and health benefits of weight training (e.g., ‘doctor’s recommendation’).

#### Weight training history

A series of questions concerning training history and current practices (age at which weight training commenced, frequency of gym attendance generally and weight training specifically, intensity of weight training) were used to obtain a profile of the sample.

### Ethics

The study was approved by the Social and Behavioural Research Ethics Committee of Flinders University. Participants were fully informed about the nature and intent of the study, with consent assumed by the return of a completed questionnaire.

## Results

Questionnaires were completed by 101 men (mean age = 30.6 years, *SD* = 9.3, range 18-67). Participants had an average weight training history of 10.1 years (*SD* = 8.3). Most indicated gymnasium attendance at least a few days a week (70.3%), with weight training most days they attended (88.1%), conducted at a moderate to moderately high intensity (72.1%) for between 30 and 90 minutes (70.3%) One third (34.3%) reported not being involved in a romantic relationship. The sample was relatively well educated, although 23.3% reported only secondary education or lower, with 21.2% reporting a trade qualification. Summary statistics for other study variables are shown in Table 1. A total BDS score was also computed, solely to derive a putative classification of dependence, defined as greater than one standard deviation above the mean (n = 18, 17.8%).

### Motives for weight training

A maximum likelihood (ML) factor analysis was used to determine whether logical clusters of motives could be identified. Maximum likelihood allows generalisation from a sample to a population (Gorsuch, 1983) and correlations with more unique variance and less error variance are given more weight (Kim & Mueller, 1985). The assumption of normality required by ML was first tested, and found to be satisfied, by inspecting the skew and kurtosis of the measured variables (Fabrigar, Wegener, MacCallum & Strahan, 1999). Second, given that items with low communalities are unlikely to contribute to factors, such items were removed from the correlation matrix until all items contributed to a putative factor. This procedure resulted in 15 of the 21 items remaining. Oblique rotation with oblimin criterion of *δ* = 0 was used to determine final factor membership given the expected theoretical overlap of derived factors. Based on parallel analysis criteria (Lautenschlager, 1989), scales were derived from three factors, accounting for approximately 53% of the variance (KMO = 0.73, Sphericity = 504.67, *p<* .001). The pattern matrix informed the potential composition of scales. Items were assigned to a scale according to their highest factor loading. Motives identified were termed personal challenge (5 items), physique anxiety (5 items) and mood control (5 items). Higher scores indicate greater endorsement of the motive. Scale membership and factor loadings are shown in Table 2.

### Concomitants of bodybuilding dependence

There was no evidence of associations between dependence and relationship status. Those with and without a current re-lationship had similar mean scores for all dependency measures. Using the dichotomous variable derived from the BDS, there was no greater likelihood of those without a current romantic relationship being dependent (x^2^(_1_) = 0.03, ns). Similarly, there was no evidence that education (x^2^(_2_) =1.54, *p* = ns), age (t(_97_) = 0.23, ns), or years of weight training (t(_92_) = 0.25, ns) were associated with dependence. Equivalent results were obtained using individual BDS scale scores.

**Table 1.** Summary statistics for key study variables

	Theoretical range	Obtained range	*N*	Mean	*SD*	*a*
Bodybuilding dependence						
Social dependency	4-28	4-28	101	12.1	5.5	.79
Training dependency	3-21	3-21	101	12.5	4.8	.76
Mastery	2-14	2-14	101	7.8	2.9	.75
Motives for bodybuilding						
Personal challenge	5-25	8-25	101	20.6	2.9	.76
Physique anxiety	5-25	5-24	101	16.4	3.7	.73
Mood control	5-25	10-25	101	18.3	3.3	.69
Perceived stress	10-50	12-46	101	25.4	6.1	.88
Anger, hostility, aggression						
Physical aggression	9-45	9-45	99	19.9	8.1	.89
Verbal aggression	5-25	6-25	99	14.1	3.7	.74
Anger	7-35	7-35	99	16.1	6.4	.89
Hostility	8-40	8-40	99	16.9	6.7	.87

**Table 2.** Factor loadings for weight training motives showing scale membership in bold

Motive item	Personal challenge	Physique anxiety	Mood control
To challenge myself	.88	-.06	-.07
For enjoyment	**.87**	.03	-.04
To maintain muscle mass	**.52**	.25	.12
To increase self-confidence	**.35**	-.01	.32
To put on muscle mass	**.30**	-.14	.10
To lose weight	-.09	**.75**	.08
To manage current weight	.16	.68	.26
To become more attractive	.15	**.56**	-.06
Peer pressure	-.17	**.51**	-.08
To avoid feelings of guilt	.17	**.45**	.05
To relieve stress	.01	.07	**.85**
To improve mood	.32	.14	**.49**
To escape daily worries	-.03	-.03	**.45**
To rid anger, aggression	-.11	.03	**.45**
Relaxation	.38	-.13	**.43**
Eigenvalues	4.13	2.08	1.74
% variance	27.52	13.88	11.59

Social dependency was most highly related to personal challenge (Table 3), with training dependency strongly associated with all motives. Mastery was only related to physique anxiety. All measures of dependence were positively associated with perceived stress. Higher levels of both anger and hostility were associated with dependence, while there was less consistent evidence that either physical or verbal aggression was related to dependence, with associations only between social dependency and physical aggression, and training dependency and verbal aggression.

### Correlates of weight training motives

Personal challenge was positively associated with other motives, but physique anxiety shared no significant association with mood control. Motives were unrelated to relationship status, education, age, or years of weight training. Similarly, little variance was shared between motives and other variables, with positive correlations only between physique anxiety and perceived stress, and mood control and hostility (Table 3).

### Correlations among other study variables

All measures of anger, hostility and aggression shared a predictable set of associations, as did the three measures of bodybuilding dependence. Correlations were also noted between anger, hostility and aggression, and perceived stress (Table 3).

### Multivariate analyses

**Figure 1.**
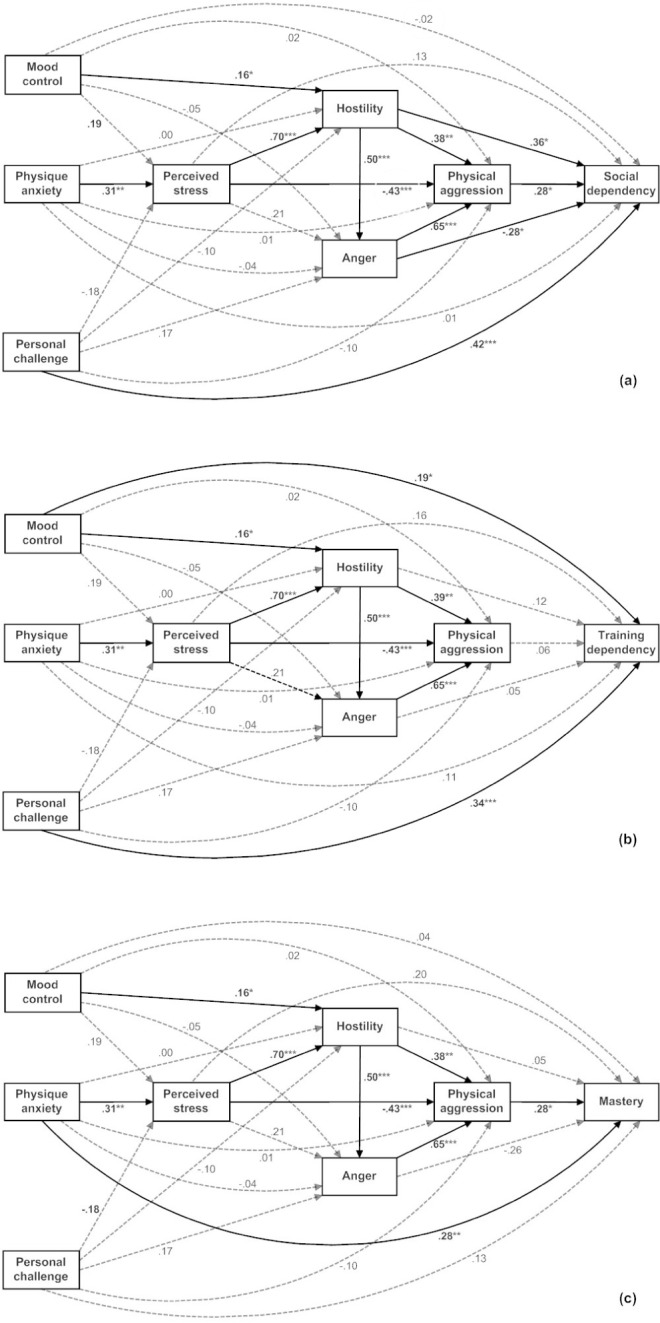
Path diagrams with beta coefficients demonstrating significant associations among study variables leading to (a) social dependency, (b) training dependency, and (c) mastery, respectively. ——— significant paths; – – – – – – non-significant paths

Acknowledging the above correlations, and the transactional model noted by Smith et al. (2004), an attempt was made to construct putative causal models to further explain the above relationships. Analyses comprised simultaneous linear regressions using a path analysis paradigm. It is acknowledged that multivariate analyses such as these are routinely conducted with structural equation modelling (SEM) techniques. However, SEM was not used, as the appropriate testing of such models requires a minimum number of participants, commonly considered to be 200 (Barrett, 2007). Bodybuilding dependence, in terms of social dependency (a), training dependency (b) and mastery (c), was considered the outcome, with three levels of predictor: (1) hostility, anger, physical aggression, (2) perceived stress, (3) motives for bodybuilding. The placement of motives in the models was selected to enable their contribution to dependence to be evaluated over and above other predictor variables (i.e., after shared variance was removed from the relevant equations). Individual models for social dependency, training dependency and mastery are presented (Figure 1). Models involving verbal aggression were not explanatory and are not presented.

#### The role of weight training motives in bodybuilding dependence

Similar, yet distinct, contributions were made by each motive to dependence. An indirect effect was noted for mood control by way of hostility in the case of all dependency measures. An additional direct effect was noted for training dependency. Physique anxiety made an indirect contribution to both social dependency and training dependency through perceived stress, while an additional direct contribution was observed for mastery. Personal challenge contributed directly to both social dependency and training dependency, but was not related to mastery.

**Table 3.** Intercorrelations among key study variables

	1	2	3	4	5	6	7	8	9	10
1. Social dependency	-									
2. Training dependency	.65^***^	-								
3. Mastery	.57^***^	48^***^	-							
4. Personal challenge	.35^***^	.43^***^	.20	-						
5. Physique anxiety	.22^*^	.34^***^	.37^***^	.36^***^	-					
6. Mood control	.22^*^	.39^***^	.15	.30^**^	.17	-				
7. Perceived stress	.29^**^	.35^***^	.24^*^	-.01	.28^**^	.19	-			
8. Anger	.23^*^	.33^***^	.13	.11	.16	.16	.54^***^	-		
9. Hostility	.39^***^	.34^***^	.22^*^	-.06	.19	.27^**^	.73^***^	.61^***^	-	
10. Physical aggression	.27^**^	.19	.19	-.04	.03	.11	.21^*^	.64^***^	47^***^	-
11. Verbal aggression	.13	.26^**^	.13	.06	.11	.19	.28^**^	.68^***^	43^***^ 59^***^	

* *p* < .05;

** *p* < .01;

***p < .001.

#### The roles of perceived stress, anger, hostility and aggression

Perceived stress was strongly positively associated with hostility, but interestingly shared a strong negative association with physical aggression (contrary to the results of bivariate analyses). Hostility was strongly predictive of anger, while both hostility and anger shared strong positive associations with physical aggression. This cluster of constructs then predicted dependence in subtly different ways. For social dependency, the effects are through physical aggression, with an additional direct contribution from hostility and anger. Training dependency was unrelated to these constructs, with the significant predictors of training dependency limited to mood control and personal challenge. For mastery, there were no direct effects attributable to predictor variables beyond physical aggression.

## discussion

This study was predicated on the assumption that bodybuilding dependence is a specific, identifiable form of the general phenomenon of exercise dependence (Adams & Kirkby, 2002; Annett et al., 1995). While the current sample of undifferentiated males engaged in regular weight training does not allow a valid commentary on precise incidence figures for bodybuilding dependence, an impressive variability of scores was evident among the sample, with at least one participant recording the maximum score for each of social dependency, training dependency and mastery. The basic proposition that there would be positive and significant associations between bodybuilding dependence and anger, hostility, aggression, and stress, was largely supported at the bivariate level. While the pattern of relationships between the measures was not consistent, the evidence was suggestive of the potential relevance of these constructs to the dependent individual.

A unique advantage of the current study was the evaluation of motives for bodybuilding. From a targeted list of 21 possible reasons for weight training, three plausible motives were derived: personal challenge, physique anxiety and mood control. Of these, mood control shared the strongest positive associations with dependence. This is particularly salient given the study’s focus on anger, hostility and aggression. Interestingly, mood control was directly associated with hostility only, suggesting they may both be cognitive self-evaluations. Further, the placement of these motives in the multivariate models allowed a commentary on their unique contribution to dependence, after shared variance with other predictors had been accounted for.

The variable pattern of results provided an opportunity to comment on the discriminant validity of the dependence constructs. While social dependency assesses the desire to be in the bodybuilding environment, training dependency literally reflects dependence on the activity, and mastery distinguishes the desire to be in control of the training schedule (Hurst et al., 2000; Smith & Hale, 2004, 2005; Smith et al., 1998). In the current study social dependency was particularly relevant to variables such as personal challenge, hostility, anger and physical aggression. These findings concur with those of Smith et al. (1998) who underlined the link between social dependency and perceptions of self-worth. In a similar vein, training dependency was associated with both personal challenge and mood control. Perhaps these associations highlight the compulsive, driven behaviour of training dependent persons. In contrast, mastery was the only dependence category associated with physique anxiety. This suggests that mastery taps a distinctly unique component of exercise dependence compared with social and training dependency (Hurst et al., 2000). Nevertheless, based on the current data the overarching conclusion must be that there are more commonalities among the three dependence subscales than differences between them. It may be judicious therefore for future studies to consider measurement models that offer more discriminating quantifications of these constructs.

There was no evidence within the current sample that individuals reporting dependence were unique in terms of relationship status, education level, age, or years engaged in bodybuilding. Some of these results are contrary to those documented by Smith and Hale (2005). It is possible that these variant results are attributable to participant characteristics. Based on the description given for the previous sample (Smith & Hale, 2005), the current participants are likely to have been better educated, with a greater proportion in a steady relationship. Further, the comment has been made that much of the research regarding bodybuilding dependence has been invariant with regard to culture (Hurst et al., 2000). While the current study contributes to this deficit, the results may simply reflect the sociocultural idiosyncrasies of the Australian male (Huggins, 1998; Taylor, Stewart & Parker, 1998). Indeed, the very decision to study only males (Hurst et al., 2000; Smith & Hale, 2005) may also offer a source of variance concerning the potential correlates of bodybuilding dependence. More generally, there is the possibility of selection bias among our participants as they were a sample of convenience. Future research may therefore benefit from the use of a control group of non-dependent weightlifters or exercisers, to enable a more effective comparison of demographic characteristics.

While it was not possible to infer causal relationships among the variables measured due to the cross-sectional nature of the study, it was nevertheless appropriate to consider the potential for causality. This was driven both by the desire to provide a framework for hypothesis testing in future research, and to acknowledge the transactional hypothesis (Smith et al., 2004) which emphasises stress disposing towards hostility and anger, to the manifestation of outward aggression, potentially leading to dependence. On balance, with the exception of training dependency, the current models reflect this proposition. This is characteristic of the transactional model (Smith et al., 2004), but not necessarily exclusive to bodybuilding or bodybuilding dependence. A unique path in our models was, however, the negative coefficient between stress and physical aggression. This should be viewed with caution as it is quite possibly a statistical artefact arising, for example, from the multicollinearity among predictor variables (e.g., stress and hostility). An alternative proposition is perhaps that weight training provides an appropriate and socially acceptable avenue to express aggressive tendencies, thereby attenuating feelings of stress. It may also be the case that engagement in physical exercise becomes a coping skill, which may contribute to, or be a reason for, dependence (Berczik et al., 2012). Future research may benefit from investigating the contribution of more specific coping skills on dependence, such as problem-focused or emotion-focused coping.

As stated previously, the models discussed above are not a commentary on the way in which the measured constructs definitively co-relate, but rather represent a platform from which alternative models, perhaps with samples of varying composition, might be tested. Indeed, the order of the predictors we have examined is somewhat interchangeable. We reiterate that the data presented are designed to generate hypotheses rather than to evaluate definitive hypotheses. Future research might reasonably evaluate alternative causal orders and/or compare associations between anger, hostility, aggression, and stress among competitive bodybuilders, amateur weightlifters and persons who exercise for general fitness (Smith et al., 1998). Future studies may also usefully include both pre- and post-training measures of these variables, along with physiological markers such as serum endorphin levels (Hamer & Karageorghis, 2007). Such designs provide the opportunity to explore the proposition that weight training improves mood, and decreases stress levels. Indeed, unless such complex longitudinal studies are undertaken, the true causal nature of the relationships considered in the current study will not be definitively understood.
